# Extraordinary abilities, creativity, and love, in the path of dementia: a perspective article on the Walking the Talk for Dementia 2025 initiative

**DOI:** 10.3389/frdem.2026.1797005

**Published:** 2026-04-13

**Authors:** Madalena P. Liougas, Prabha Shrestha, Belen Custodio, Lenisa Brandao, Kevin Cullen, Therese Maher, Karen Young, Alan Young

**Affiliations:** 1 Temerty Faculty of Medicine, Rehabilitation Science Institute, University of Toronto, Toronto, ON, Canada; 2 KITE Research Institute, Toronto Rehabilitation Institute-University Health Network, Toronto, ON, Canada; 3 Department of Nursing and Midwifery, Kathmandu University School of Medical Sciences, Dhulikhel, Nepal; 4 Global Brain Health Institute, San Francisco, CA, United States; 5 Research Department, Instituto Peruano de Neurociencias, Lima, Peru; 6 Speech, Language and Hearing Department, Federal University of Santa Maria (UFSM), Santa Maria, Brazil; 7 The Dementia Research Advisory Team (PPI), Alzheimer Society of Ireland, Dublin, Ireland; 8 Walking the Talk for Dementia, Belo Horizonte, Brazil

**Keywords:** collaboration, connection, dementia, dementia-friendly, meaningful engagement, person-centered, policy

## Abstract

This manuscript, co-created by researchers and people living with dementia and their care partners, explores how engagement with lived experience can transform conventional, deficit-based understandings of dementia—often defined by what is lost. While clinically useful, this framing can obscure the richness, resilience, and relational depth present in the lives of people living with dementia. Drawing on a collaborative and reflective process inspired by participation in Walking the Talk for Dementia 2025—a global immersive event that brought together people with lived experience of dementia, researchers, advocates, clinicians, and artists—the paper challenges traditional narratives of dementia. Through in-person and virtual discussions and documented reflections, the team conducted a qualitative thematic analysis to identify key insights. Three themes emerged: (1) ‘Dementia Reimagined’, highlighting personal and professional transformation and new identities; (2) ‘Flourishing through Creativity and Resilience’, illustrating humor, creativity, leadership, and authentic connection; and (3) ‘Dementia as a Catalyst for Love and Understanding’, emphasizing enduring love and deepened empathy within families and communities. These findings demonstrate that living with dementia can foster resilience, creativity, and meaningful human connection. This experiential, co-produced approach reframes dementia beyond deficit-based models, offering transformative insights that can inform person-centered care practices, policy development, and increase awareness.

## 1 Introduction

Throughout the past two decades, dementia policy has undergone a global shift from a deficit-based perspective highlighting decline, disorder, and loss to one that emphasizes the capability of people with dementia to live well, a perspective grounded in social models of disability and aging (Quinn et al., 2022; Putnam and Stark, 2025). In 2012, the World Health Organization (WHO) designated dementia as a public health priority, prompting many countries to develop national dementia plans (Organisation mondiale de la santé, 2012; World Health Organization, 2017). Many national dementia plans address areas such as increased awareness, improved care and treatment, person-centered care, and quality of life (Alzheimer Europe, 2018; Australian Government D of AC, 2024; Department of Health (UK), 2009; Ministry of Health, Welfare and Sport (Netherlands), 2021; Public Health Agency of Canada and WHO, 2019; Parra et al., 2021). To implement the goals of national dementia plans, it is essential to involve people with lived experience of dementia in the research and policy-making processes (World Health Organization, 2017; Snowball et al., 2022; Vijayasingham et al., 2025; Litherland et al., 2018).

Clinically, dementia is an umbrella term encompassing a range of conditions marked by cognitive decline that interferes with daily functioning (American Psychiatric Association, 2013). As dementia progresses, individuals may experience structural changes in the brain that affect memory, reasoning, and behavior (American Psychiatric Association, 2013). Yet research suggests that quality of life does not necessarily diminish in the presence of dementia, especially when adequate support systems are available (Hoe et al., 2009; Clare et al., 2022). Relationship factors, such as social connectedness and engagement, are associated with quality of life, making them key targets for research, interventions, and policies addressing dementia (Martyr et al., 2018; O’Rourke et al., 2015).

Relationship factors are influenced by an individual’s socio-demographic, socio-economic, and health characteristics (Lampinen et al., 2022; Cohen-Mansfield et al., 2016; Fernández-Mayoralas et al., 2015). Collectively, these characteristics can thus act as either facilitators or barriers to adequate support systems and, ultimately, to the ability to live well with dementia. According to the World Alzheimer Report (Gauthier et al., 2022), many individuals live in areas where support systems for post-diagnostic care are unclear, insufficient, or non-existent, often characterized by limited healthcare infrastructure, low availability of dementia specialists, economic constraints, and cultural or systemic barriers that hinder access to care (Migeot et al., 2025; Ibáñez et al., 2021). These systemic inequities underscore the need for inclusive, participatory research that centers people with dementia and care partners as co-creators of knowledge, ensuring that support and policies are responsive to local contexts and lived realities (Reyes et al., 2023; Rojas-Rozo et al., 2024).

To address these challenges, the third annual Walking the Talk for Dementia (WTD) was held in August 2025 in Santiago de Compostela, Spain, to strengthen relationships, overcome geographic barriers, and promote living well with dementia (Aguzzoli Peres et al., 2024). A total of 85 people from over 25 countries, including people with dementia, their care partners, healthcare professionals, researchers, policymakers, and advocates, walked 40 km along the Camino de Santiago pilgrimage route, followed by a two‐day symposium. WTD aims to advance global dementia policies by demonstrating that, with supportive environments, people with dementia can continue to engage in meaningful activities, relationships, and shared learning (Bechard et al., 2022).

This manuscript emerges from such a collaborative ethos. Researchers and people with lived experience of dementia worked together to reflect on and reframe dominant discourses of dementia at WTD 2025. We aim is to demonstrate how participatory engagement can illuminate new dimensions of dementia, fostering more inclusive and compassionate approaches to care, research, and policy.

## 2 Approach

This manuscript adopted a co-production approach between people with lived experience of dementia and researchers who participated in WTD 2025 (Aguzzoli Peres et al., 2024; Bourke et al., 2024). Four researchers and four people with lived experience from four continents (Europe, North America, South America, and Asia) participated. Researchers brought expertise in dementia care, social sciences, and public health; people with lived experience contributed perspectives as persons living with dementia and care partners. This diversity ensured multiple viewpoints informed the analysis.

The manuscript draws on three sources of co-produced input:

(1) In-person dialogues at WTD 2025 (Santiago de Compostela, Spain, August 24–31, 2025), including two structured small-group discussions (60–75 min each) convened during the walk and symposium (held over 6 days).
(2) Virtual discissions, consisting of three synchronous 90-min video meetings held over Zoom held between November 2025 and January 2026, scheduled to accommodate participants across time zones.
(3) Independent reflections, with each author submitting one reflection (eight in total) in either written format (500–1,000 words; *n* = 7) or short video format (45 min; *n* = 1).

All sources of input were oriented around the shared guiding question, ‘How can engagement with people with lived experience of dementia transform conventional, deficit-based understandings of dementia and inspire new perspectives on what it means to live with dementia?’ Sub-questions explored: (a) experiences of capability, agency, and interdependence; (b) social and environmental conditions that enable participations; (c) implications for dementia research, care and policy. It is important to note that the reflections stimulated by this question were anchored in the experiences, conversations, and encounters that took place within and around WTD 2025, while acknowledging that participants’ broader lived experiences and daily realities also inevitably informed their contributions.

In-person and virtual discussions were documented using contemporaneous note-taking by MPL, PS, BC and LB. Audio and video recording were not used for in-person or virtual group discussions to support open-exchange and accessibility among the research team. Notes captured body language, illustrative examples, and areas of convergence or divergence. Independent reflections were submitted as text documents or video files with transcripts, which were compiled into a shared and secure OneDrive folder, which served as the shared workspace.

Data analysis followed a rapid, pragmatic, and co-produced thematic synthesis appropriate for a perspective article. First, MPL, PS, BC and LB conducted open coding across all materials, identifying recurring concepts and clustering them into preliminary categories. Second, an initial memo of shared meanings and candidate themes was circulated to all co-authors for review, elaboration, and critique. Third, themes were refined through two iterative consensus meetings (60 min each), during which areas of agreement, tension, and emphasis were discussed and resolved. Divergent interpretations were retained where relevant and informed the final thematic framing (Braun and Clarke, 2006).

People with lived experience were involved across the research process, including determining the guiding question, generating materials (independent reflections), refining themes, and shaping the manuscript text. First, all WTD 2025 participants with lived experience were invited to join the research team. Second, participants who indicated their interest were asked to reflect on the WTD 2025 event; based on these reflections, the researchers (MPL, PS, BC and LB) posed the guiding question, whereby people with lived experience provided feedback and the question was refined. Third, everyone (researchers and people with lived experience) submitted an independent reflection. The researchers then invited each person with lived experience to contribute to the generation of themes, however, everyone with lived experience stated that they preferred to contribute to the refinement of themes. Thus, the researchers determined a set of preliminary themes and shared them with people with lived experience who provided written and oral feedback. The final manuscript was then shared with people who have lived experience, and they again provided written and oral feedback.

## 3 Three emergent themes derived from collaborative reflections regarding the reimagination of dementia through the lived experience in WTD 2025

Our collaborative reflections revealed perspectives that reframe the possibility of living with dementia with meaning, creativity, and connection. Three overarching themes emerged from our analysis (Table 1).

**Table 1:** An overview of the three themes determined from the qualitative analysis.

Theme	Description
(1) Dementia reimagined	Describing shifts in identity and the dismantling of hierarchical power structures
(2) Flourishing through creativity and resilience	Illustrating adaptive strategies, leadership, and new ways of connecting
(3) Dementia as a catalyst for love and understanding	Underscoring the enduring presence of love and empathy within families and communities

### 3.1 Theme 1: dementia reimagined

Individuals challenged clinical and deficit-based narratives by reframing dementia as a lived experience shaped by resilience, identity, and purpose. People living with dementia, care partners, and healthcare professionals recognized that while some abilities change, new ways of expressing, leading, and connecting can emerge. Many, especially those in healthcare roles, had been trained to prioritize what is lost. However, people with dementia revealed something different: the condition does not simply narrow life but can open multiple possibilities when people are treated with respect, their needs are prioritized, and they are genuinely listened to.

For those with dementia and their care partners, WTD offered a chance to reclaim identity after diagnosis. Instead of viewing it as an ending, they reframed it as a beginning, a shift that created space for advocacy, new passions, and renewed purpose. The diagnosis became part of their story, not the entirety of it, acknowledging that a lifetime of experiences remains intact.

#### 3.1.1 Transformative growth

Care partners expressed joy in realizing how much they had grown in knowledge, confidence, and acceptance: “Through learning and shared experiences, I understand more deeply how dementia affects the person I love, and I am more determined than ever to raise awareness of Lewy Body Dementia. I carry the strength to continue being a warrior and a champion for all who live with this condition.”

On the same line, healthcare and social care professionals described being transformed by this encounter, gaining an experiential understanding that no textbook or lecture could offer. This challenged them to confront previously unnoticed prejudices: the subtle ways they reduced people to symptoms or overlooked their capacity for agency and contribution.

#### 3.1.2 Expanding the narrative

A collective shift emerged: moving beyond centering only challenges, not because difficulties do not exist—they certainly do—but they do not capture the whole reality. Dementia became more than a medical label, a metaphor for unity and shared commitment. People living with dementia possess extraordinary abilities and are experts in their own experience. “Nothing about us without us” emphasized placing their voices at the heart of every conversation and change. One person shared: “I live in the present moment. I have dementia, which is difficult, but I am still alive and in touch with reality. Even if I forget the moment that just passed, I am once again present.”

This process was supported by the atmosphere created during WTD, a space where people moved forward together, literally and symbolically, with a sense of unity and purpose. The event created a space where people moved forward together, united and mutually recognizing one another. Being embraced by a community offering understanding without judgment allowed new identities to emerge. Reframing dementia means expanding the narrative to include strength, resilience, and transformation—seeing the person with the life experience they have always had before the diagnosis and recognizing that with support, people living with dementia can continue to lead and find renewed meaning.

### 3.2 Theme 2: flourishing through creativity and resilience

This theme captures how people with dementia, care partners, and professionals expressed creativity and resilience throughout WTD. Creativity appeared both as a mode of adaptation and a relational practice, while resilience manifested through flexible strategies, emotional steadiness, and shared support.

#### 3.2.1 Adaptive strategies and environmental attunement

People with dementia described resilience as a dynamic set of adaptive practices that enabled them to remain engaged and connected. One person emphasized the importance of staying open rather than withdrawing (“Don’t hide away”) and outlined sensory-regulation strategies, including seeking quiet spaces and using noise-canceling headphones to manage overwhelming environments. These everyday adjustments represent practical creativity, enabling continued participation while preserving comfort.

Creativity also appeared in relational practices characterized by presence, reciprocity, and non-hierarchical connection. A person with frontotemporal dementia articulated this through the insight: “Being with is far more important than Doing to.” This perspective reflects a creative relational stance in which meaningful connection arises through presence rather than intervention, challenging care models that prioritize action.

Care partners’ reflections further reinforced this orientation. Subtle gestures such as steadied hands, pauses for rest, and moments of quiet understanding functioned as forms of relational creativity, strengthening resilience through companionship. These interactions emphasized that resilience can be cultivated through relational presence, offering individuals a sense of grounding and shared humanity. Across accounts, relational creativity emerged as a foundational dimension of resilience, enabling people with lived experience to navigate uncertainty without feeling isolated.

#### 3.2.2 Artistic practices as internal regulation

Creativity also served as a mechanism for internal regulation. A person with frontotemporal dementia described moments of severe cognitive overload, “my bursting dementia brain… ready to explode” that shifted abruptly into calm through an improvised artistic ritual. Painting a heart-shaped stone and repeatedly layering paint even after the object was complete became a rhythmic sensory practice that stabilized her mental state. The repetitive motion served as a grounding mechanism, demonstrating how artistic engagement can support emotional modulation and cognitive equilibrium.

A care partner similarly described the art workshop as an “inward pilgrimage,” where brushes, colors, and shared laughter reawakened long-dormant impulses to create. Within this safe, communal environment, creativity resurfaced as a pathway to vitality and expression. Both accounts highlight creative reawakening as a significant element of resilience for people living with dementia and their care partners.

#### 3.2.3 Identity work and existential resilience

Resilience also emerged through identity work and the capacity to confront vulnerability with clarity. A person with frontotemporal dementia expressed existential concerns regarding possible changes in her inner empathy, describing it as “the core of who I am.” Her reflection exemplified an inward form of resilience grounded in agency, lucidity, and self-understanding.

A researcher trained in rehabilitation sciences described how the walk’s inclusive environment enabled them to reconnect with her own history as a care partner. The experience helped them process suppressed emotions and understand the roots of their professional purpose, illustrating how resilience can arise through integration of personal and professional identities. Together, these reflections show identity not as static but as continually reorganized through acceptance and creative adaptation.

#### 3.2.4 Belonging and emotional resilience

Emotional resilience was reinforced by openness, gratitude, and a sense of belonging. One person living with dementia described how being among others who care about dementia fostered hope, emphasizing mutual learning and engagement (“Learn from others. Help others.”). These reflections highlight relational generosity and shared humanity as core components of resilience.

Care partners described the walk as an environment of genuine companionship and emotional healing. They felt safe enough to let down their inhibitions, reconnect with themselves, and experience renewed confidence. Their choice to mark the experience with a tattoo of the pilgrim’s shell—an action shared by several WTD participants—became an embodied expression of resilience. The tattoo functioned as a communal anchor, symbolizing continuity, belonging, and transformation. Across these accounts, emotional resilience was strengthened through belonging, embodied expression, and openness within a supportive community.

### 3.3 Theme 3: dementia as a catalyst for love and understanding

This theme explores how love emerged as a force unaltered by dementia, deepening empathy, dignity, and relational closeness within families and communities.

#### 3.3.1 Love as an enduring force beyond cognitive change

Across the many stories shared during the WTD journey, one message consistently emerged: love does not disappear when dementia enters a person’s life. The diagnosis may shift routines and alter behaviors, but it cannot erase the person at the core. Another person captured this truth with clarity: “Even after the diagnosis of dementia, the actual person is still in there, and to treat them accordingly is very important.” Despite the behavioral and emotional shifts that dementia may cause, relationships often grow stronger. As one practitioner reflected, “I understood that dementia has no power over love.” These words challenge how dementia is typically feared for what it takes away, revealing instead what can never be taken: love, presence, and human worth. Affection is sustained not by perfect communication but by emotional presence as relationships evolve, deepening empathy, patience, and closeness between care partners and people living with dementia.

The essence of WTD can be summed up in one powerful word: love. One person captured this beautifully: “Love was present in every interaction.” In the gentle care between family members, in partners looking out for one another, and in the warmth shared among all who took part, love manifested not as an abstract idea but as something lived and felt in ordinary moments together. For this person, love was inseparable from trust and safety: “To feel loved, trust and a safe space are essential; this space and WTD have created that space.” This reflection brings the theme full circle, dementia can unsettle families, strain routines, and test everyone’s patience, but it can also sharpen the focus on what truly matters.

#### 3.3.2 Self-love, acceptance, and inner peace

One person with frontotemporal dementia offered a deeply moving picture of courage and self-acceptance. She introduced herself with warmth and assurance, naming her diagnosis without flinching and refusing to let it define her. She spoke about walking this path without a partner and still being able to say, “it was all right”, not as a denial of loneliness but as an expression of inner steadiness and internal trust. Her acceptance of the situation, especially her choice to let a friend who loved her not “give up their life” for them, revealed striking emotional clarity. Rather than clinging out of fear, she chose to protect the other person’s freedom, showing that love sometimes means loosening one’s grip rather than tightening it. In that decision lies autonomy: she is not only someone who needs care but also someone still capable of caring for others’ wellbeing and future.

#### 3.3.3 Reciprocal care and mutual support

The walk became a space where compassion flowed naturally. Unspoken needs were met: a steadying hand, a pause for rest, watermelon and juice to restore energy. These gestures demonstrated that support deepens rather than diminishes autonomy. One researcher described an encounter with a care partner who felt not just like a caregiver but also cared for. This emphasized that people living with dementia can still care for and love their partners.

Dementia may alter life’s course but not a person’s essence. It often deepens love, patience, and mutual understanding. Dementia serves as a catalyst for human connection, motivating families to love more profoundly and face the journey together with courage and hope. Participants witnessed extraordinary abilities, safe spaces, and the possibility of renewal after diagnosis, underscoring the urgent need to challenge stigma.

## 4 Discussion

This perspective article presents a co-produced analysis of lived experiences from WTD 2025, revealing how participatory engagement can transform conventional understandings of what it means to live with dementia. Rather than conceptualizing dementia primarily as a trajectory of loss, the findings position dementia as a relational, meaning-making experience shaped by social context, opportunity, and connection. Three interconnected themes emerged: dementia reimagined (identity transformation and empowerment), flourishing through creativity and resilience, and dementia as a catalyst for love and understanding.

The findings align with and extend existing literature on what it means to live well with dementia (Quinn et al., 2022; Clare et al., 2019; Clare et al., 2014). The first theme, Dementia Reimagined, resonates with person-centered care frameworks that emphasize identity, agency, and social citizenship rather than solely focusing on cognitive decline (McGilton et al., 2012; Mohr et al., 2021). Participants’ experiences of reclaiming identity after diagnosis and discovering renewed purpose support research indicating that quality of life can be maintained or even enhanced when individuals receive adequate support and are treated with dignity (Banerjee, 2006; Banerjee et al., 2009). The shift described by healthcare professionals, viewing dementia through a deficit lens to recognizing capacity and contribution, reflects the broader paradigm shift called for in global dementia policy. The findings also suggest that identity reconstruction does not occur solely at the individual level; rather, it is co-produced through social encounters that validate capacity, contribution, and belonging (Fuchs, 2020). This has implications for dementia research and practice, which is beginning to recognize the importance of relationship-centered care rather than functional outcomes in living with dementia (Nolan et al., 2004; Soklaridis et al., 2016). The second theme, Flourishing through Creativity and Resilience, complements existing empirical literature demonstrating that interventions targeting creative expression can help people with dementia to connect with themselves and others (Corbin et al., 2021). Descriptions of sensory regulation, environmental attunement, and artistic practices are mechanisms for maintaining engagement, align with evidence that creative activities such as music, clowning, and dancing can support emotional wellbeing for people with dementia and those who support them (Smith et al., 2021; Hendriks, 2012; Kontos et al., 2021). The concept of “being with” rather than “doing to” challenges task-oriented care models and supports relational approaches (Tieu and Matthews, 2024). Resilience in dementia is not an individual trait, but a relational process co-constructed through supportive environments and meaningful connections. This reframing has important implications for how resilience is operationalized in dementia research and how support services are designed and evaluated, it is important for services to be strength-based, person-centered and facilitate reciprocal social interactions (Whelan et al., 2020).

The third theme, Dementia as a Catalyst for Love and Understanding, extends understanding of relationship quality in dementia care. While some literature focuses on caregiver burden and strain (Banerjee et al., 2009), participants emphasized how the presence of dementia can also deepen empathy, strengthen bonds, and clarify what matters most in relationships (Kovaleva et al., 2018; Eskola et al., 2022). The reciprocal nature of care described by participants where people with dementia continue to care for their partners disrupts unidirectional models of caregiving and recognizes the ongoing agency and relational capacity of people with dementia (Swall et al., 2020). The recognition that dementia has no power over love challenges narratives of inevitable relational decline and supports research indicating that emotional connection and attachment can remain intact even as cognitive abilities change and even as relationships evolve to take different forms as cognitive abilities change over time. For services and policy, this argues for models that intentionally scaffold reciprocity (e.g., shared decision-making, mutual support roles, dyadic interventions) rather than positioning partners solely as “carers” and people with dementia solely as “care recipients” (van der Flier et al., 2017; Falzarano and Czaja, 2023).

The findings have several important implications. From an educational perspective, health and social care training should incorporate experiential learning opportunities that bring students and professionals into authentic relationships with people with dementia, moving beyond clinical observation to genuine partnership (Falzarano and Czaja, 2023; Barnes et al., 2012; Cheung, 2023). Encounters such as those facilitated by WTD has demonstrated that direct engagement with lived experience can fundamentally shift how professionals understand dementia, challenging deficit-based assumptions that are deeply embedded in clinical training. Accordingly, a concrete next step is to embed co-taught modules led by people with dementia, longitudinal placements in community settings, and assessment of relational competence (e.g., perspective-taking, collaborative goal-setting) as core learning outcomes (Aworinde et al., 2024; Yang et al., 2025). Future curricula should therefore intentionally integrate co-learning models that recognize people with dementia as educators and experts in their own right. At a policy level, dementia policies and services must be co-designed with people with lived experience from the outset, ensuring their voices shape priorities, implementation, and evaluation (Liougas et al., 2025). Practically, this entails resourcing standing panels of people with dementia and care partners with decision rights (not only consultative input), integrating lived-experience criteria into commissioning and procurement, and reporting co-production processes and impacts transparently in strategy documents (Bethell et al., 2025). The insights generated through initiatives like WTD offer a compelling case for embedding participatory approaches not only in research but in the development and revision of national dementia strategies, particularly in regions where such frameworks are still emerging. At the environmental and community level, the WTD initiative highlights the importance of creating dementia-friendly environments that support continued participation in meaningful activities (Craig et al., 2024). Taken together, these implications point toward a broader cultural shift in dementia services, moving from managing decline to nurturing potential, and from speaking about people with dementia to building knowledge and policy alongside them. For the research agenda specifically, we propose three priorities: (1) develop and validate relational-oriented outcome measures that capture recognition, agency, and participation; (2) test co-produced, experiential learning models and evaluate impacts on professional practice and service quality; and (3) design policies that use genuine co-production to ensure successful implementation for more equitable outcomes regarding living well with dementia.

### 4.1 Limitations

Several limitations should be acknowledged. The people with lived experience involved had access to healthcare and the resources required to complete the walk. Moreover, participants were likely already engaged in advocacy, potentially limiting transferability to broader populations with dementia. Additionally, dementia is experienced in highly individual ways and this perspective does not capture how factors such as age, dementia type, or disease progression may differentially influence experiences. The reflective, co-produced methodology, while appropriate for a perspective article, differs from systematic qualitative research and may introduce shared interpretive biases.

## Data Availability

The raw data supporting the conclusions of this article will be made available by the authors, without undue reservation.
